# Beyond Pathway Analysis: Identification of Active Subnetworks in Rett Syndrome

**DOI:** 10.3389/fgene.2019.00059

**Published:** 2019-02-21

**Authors:** Ryan A. Miller, Friederike Ehrhart, Lars M. T. Eijssen, Denise N. Slenter, Leopold M. G. Curfs, Chris T. Evelo, Egon L. Willighagen, Martina Kutmon

**Affiliations:** ^1^Department of Bioinformatics - BiGCaT, NUTRIM School of Nutrition and Translational Research in Metabolism, Maastricht University, Maastricht, Netherlands; ^2^GKC-Rett Expertise Centre, MUMC+, Maastricht, Netherlands; ^3^Department of Psychiatry and Neuropsychology, School for Mental Health and Neuroscience, Maastricht University, Maastricht, Netherlands; ^4^Maastricht Centre for Systems Biology (MaCSBio), Maastricht University, Maastricht, Netherlands

**Keywords:** pathway analysis, WikiPathways, Reactome, Rett syndrome, network analysis, RDF, topology, active subnetworks

## Abstract

Pathway and network approaches are valuable tools in analysis and interpretation of large complex omics data. Even in the field of rare diseases, like Rett syndrome, omics data are available, and the maximum use of such data requires sophisticated tools for comprehensive analysis and visualization of the results. Pathway analysis with differential gene expression data has proven to be extremely successful in identifying affected processes in disease conditions. In this type of analysis, pathways from different databases like WikiPathways and Reactome are used as separate, independent entities. Here, we show for the first time how these pathway models can be used and integrated into one large network using the WikiPathways RDF containing all human WikiPathways and Reactome pathways, to perform network analysis on transcriptomics data. This network was imported into the network analysis tool Cytoscape to perform active submodule analysis. Using a publicly available Rett syndrome gene expression dataset from frontal and temporal cortex, classical enrichment analysis, including pathway and Gene Ontology analysis, revealed mainly immune response, neuron specific and extracellular matrix processes. Our active module analysis provided a valuable extension of the analysis prominently showing the regulatory mechanism of *MECP2*, especially on DNA maintenance, cell cycle, transcription, and translation. In conclusion, using pathway models for classical enrichment and more advanced network analysis enables a more comprehensive analysis of gene expression data and provides novel results.

## 1. Introduction

In a diseased state, many molecular processes in the human body are affected and dysregulated. Performing pathway analysis on molecular data sets comparing healthy vs. diseased subjects is immensely effective in finding affected pathways and it enables researchers to study the underlying processes in detail, to reveal possible disease mechanisms. While standard enrichment methods have limitations and pathways are analyzed independently with their arbitrary process boundaries (Khatri et al., 2012), the pathway models themselves are very interesting from a network science perspective. These models contain detailed information about biological molecules and their interactions with one another, which can be visualized and analyzed using network biology tools (Kutmon et al., 2014). The detailed models of these biological processes are collected in online pathway databases like WikiPathways (Slenter et al., 2017) and Reactome (Fabregat et al., 2017). The availability of pathway models in the structured and semantic Resource Description Framework format (RDF) creates the possibility to integrate all pathway models into one large network and therefore incorporate the relations and overlap between them (Waagmeester et al., 2016). By removing artificial boundaries, this will enable us to study the systemic effects of diseases, such as Rett syndrome, using network biology methods. Specifically, we can look for subnetworks, even if not present in pathways as found in pathway databases, which reflect modules of differential biological activity.

Rett syndrome (MIM: 312750, Rett, 1966) is a rare genetic disorder, caused in most patients by a loss of function mutation in the *MECP2* gene (Amir et al., 1999). The accompanying MECP2 protein is multifunctional and acts as an epigenetic repressor, transcriptional repressor, and transcriptional activator. MECP2 binds DNA on methylated CpG islands and is involved in several regulatory activities: attracting histone deacetylases (HDAC1), increasing packing density of DNA, repressing and in specific genes also activating gene expression, and due to its phosphorylation sites, MECP2 activity is sensitive to intracellular signaling (Chunshu et al., 2006; Ehrhart et al., 2016). Due to its regulatory role, many downstream genes are affected in case of loss of function, resulting in a broad range of symptoms including moderate to severe intellectual disability, gait problems, stereotypic movements, dystonia, scoliosis, epileptic seizures, and sleep problems (Hagberg et al., 2002; Neul et al., 2010). In the past 10 years, omics data analysis on the level of genome, transcriptome, or proteome saw an increase in importance, to analyse and understand the holistic impact of MECP2, respectively, the impact of an impaired MECP2. Shovlin and Tropea (2018) recently reviewed the available transcriptomics studies on Rett syndrome and came to the conclusion that the most researched impact of MECP2 dysfunction lies with dendritic connectivity and synapse maturation, mitochondrial dysfunction, and glial cell activity. Recent pathway analysis results of single and integrated studies identified changes in intracellular signaling, including EIF2 (eukaryotic translation initiation) signaling, cytoskeleton, and cell metabolism including mitochondrial function (Bedogni et al., 2014; Ehrhart et al., 2018).

In this study, we aim to investigate the molecular changes in Rett syndrome patients using a network-based approach by integrating existing pathway models from WikiPathways and Reactome into one large network and identifying disease-affected submodules that show differential gene expression. We will compare the results with standard enrichment analysis methods, including pathway and Gene Ontology analysis, and expect that the identified disease modules will also contain interactions in pathways not found through those methods.

## 2. Materials and Methods

### Dataset

The publicly available dataset studying the transcriptome in human brain tissue of Rett syndrome patients and healthy controls from the Gene Expression Omnibus (GEO) was used (GEO:GSE75303). The original study was published by Lin et al. (2016). The dataset contains transcriptome data obtained with Illumina HumanHT-12 V4.0 expression beadchips. The samples were taken postmortem from human frontal and temporal cortex of three Rett syndrome patients (*MECP2* mutations c.378-2A>G, c.763C>T, c.451G>T) and three age-, gender-, and ethnicity-matched controls.

Raw and normalized data as well as study metadata were obtained (GEO:GSE75303) and subjected to quality control, including signal distribution and sample grouping analyses, using plotting functions from ArrayAnalysis.org (Eijssen et al., 2013). No samples were excluded for further analysis. The provided normalized data on GEO was filtered to remove all probes with a detection p-value of 1 for all samples, indicating overall absence of expression. Thereafter, the limma package for R (version 3.30.13, Ritchie et al., 2015) was used to compute differential expression between Rett patients and controls for the frontal and temporal cortex samples separately. For each probe, this results in estimates of the fold change and *p*-value significance between the patient and control groups. Probes were re-annotated with Ensembl gene identifiers based on Ensembl build 91 using the BridgeDbR package (version 1.16.0, Leemans et al., 2018) with the Hs_Derby_Ensembl_91.bridge database (van Iersel et al., 2010).

### Enrichment Analysis

We performed pathway analysis with PathVisio (version 3.3.0, Kutmon et al., 2015) and Gene Ontology (GO) analysis with GO-Elite (version 1.2, Zambon et al., 2012).

For GO analysis with GO-Elite, the input gene lists for frontal and temporal cortex contained all significantly changed genes (*p*-value < 0.05) with an absolute fold change cutoff of 1.5. Ensembl identifiers of all measured genes in the datasets were provided as the background list. Number of permutations was set to 2,000. Pruned GO-term results (i.e., GO terms for which genes in subterms that were found to be significant were removed) were filtered based on Z-score (> 1.96), permuted *p*-value (< 0.05) and a minimum number of changed genes of five. Pathway analysis was performed on a combined human pathway collection from all curated WikiPathways pathways including the Reactome pathway set (in total 903 pathways, October 2018 release). Differential gene expression was mapped to genes on the pathway diagrams using the Hs_Derby_Ensembl_91.bridge identifier mapping database. Thereafter, pathway statistics was performed on differential gene expression for temporal and frontal cortex using the following criteria to select only significantly differentially expressed genes (absolute fold change cutoff of 1.5 and *p*-value < 0.05):

(log2FC < –0.58 OR log2FC > 0.58) AND *p*-value < 0.05.

The resulting ranked pathway list was filtered based on Z-score (> 1.96), permuted *p*-value (< 0.05), and minimum number of changes (positive) genes of five.

### Pathway-Based Network Construction

Biological pathway models are small sub-networks describing specific biological processes. Connecting and integrating pathway models in one large network enables us to use network biology tools and approaches to study and investigate the network.

**Figure 1:**
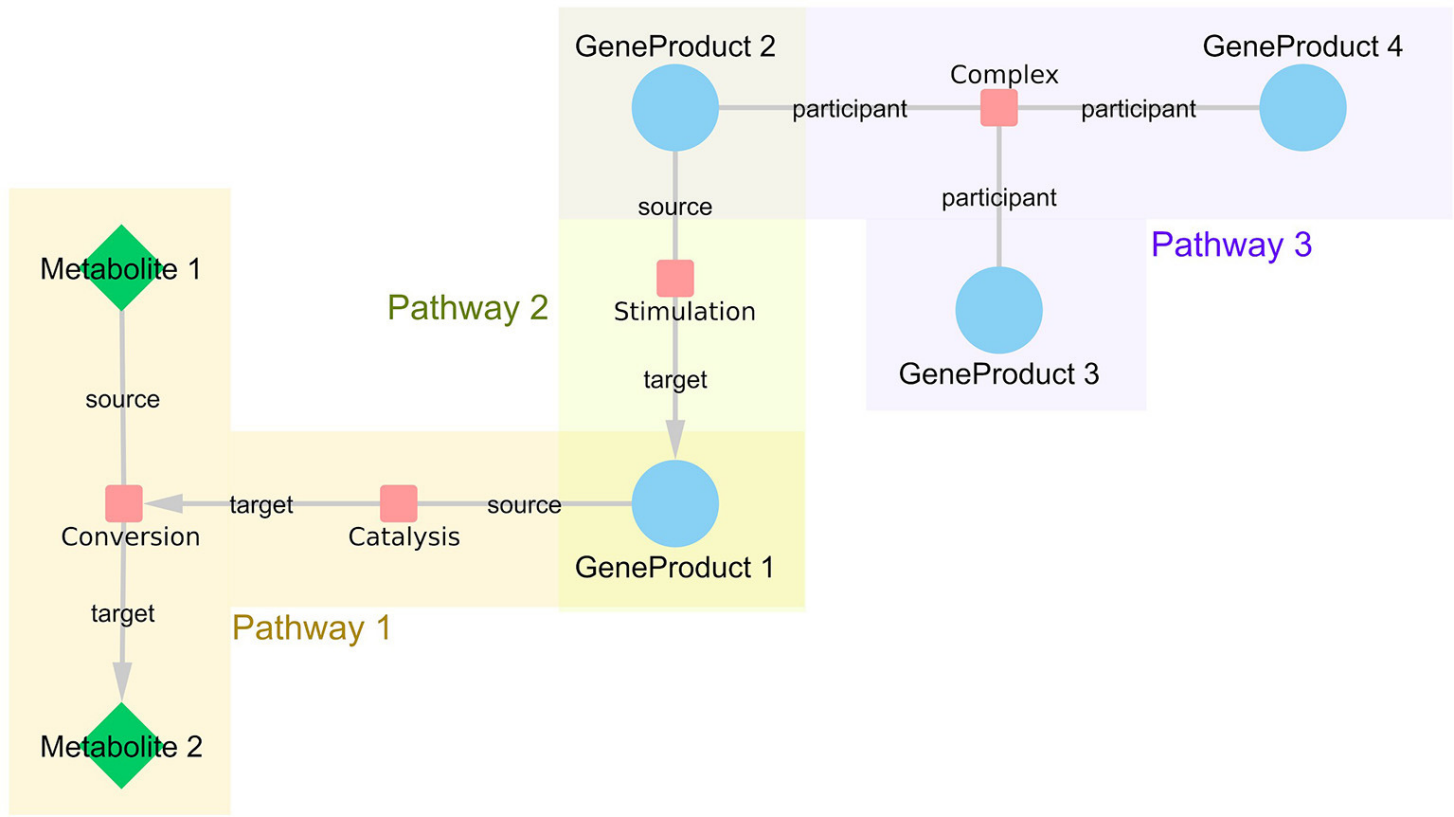
WikiPathways network structure. Every interaction is represented as a node in the network with links to all participants. If the interaction is directed, the information about source and target nodes is added as an edge attribute. The nodes represented as small, red rounded rectangles are interactions, blue circles represent gene products and green diamonds embody metabolites. Interactions that share certain participants, such as GeneProduct 1, are brought close together in the resulting network even if they are from different pathways, such as Pathway 1 and 3.

We used the WikiPathways RDF from October 2018 release (Waagmeester et al., 2016) to retrieve information about all interactions in the pathway models of two major pathway databases, WikiPathways and Reactome. With this network approach, the pathway models are not treated as independent modules, but they are integrated on an interaction level, which enables linking information from different pathways based on their shared participants and thus bringing related interactions closer to each other. As shown in Figure 1, each interaction is represented by an interaction node in the network with edges to all participant nodes (either source, target, or participant). For each interaction, it is recorded in which pathway or pathways the interaction is present. By connecting all the retrieved interactions, a large network representing all human pathway models was created. The SPARQL query language was used to retrieve the relevant data. The scripts to generate the constructed network are available on GitHub (https://github.com/wikipathways/wprdf2cytoscape). Interactions with at least two annotated interaction participants (gene product, metabolite, complex) are included. Gene products have unified Ensembl (Zerbino et al., 2017) identifiers, metabolites have either Wikidata (Mietchen et al., 2015), ChEBI (Hastings et al., 2015) or HMDB identifiers (Wishart et al., 2017), and complexes have Reactome identifiers. A list of frequently occurring small molecules (Supplementary Table 1), e.g., H^+^, H_2_0, ATP, were removed from the network to prevent inclusion of paths with no specific biological relevance. Such small molecules tend to create artificial hub nodes simply because e.g., ATP is used/produced in a lot of metabolic reactions.

### Active Module Analysis

The constructed network was loaded into Cytoscape (version 3.7.0), a network analysis and visualization tool (Shannon et al., 2003). Differential expression analysis data (log2 fold changes and *p*-values) for both frontal and temporal cortex were added as node attributes to the network.

The Cytoscape app jActiveModules (version 3.2.1, Ideker et al., 2002) was used to identify active submodules in the large network that show significant changes in expression. These subnetworks are freed from the artificial pathway boundaries of conventional pathway models found in WikiPathways and Reactome. The following parameters were used to find active submodules: *p*-value as the node attribute, number of modules was set to five, overlap threshold of 0.8, and search strategy with a search depth of two.

### Tools and Settings

- Dataset: Normalized data from GEO, plotting functions from ArrayAnalysis.org, limma package for R (version 3.30.13), BridgeDbR package (version 1.16.0) with Hs_Derby_Ensembl_91.bridge database.
- Enrichment analysis: PathVisio (version 3.3.0) and GO-Elite (version 1.2).
- Pathway-based network construction: Script available on Github(https://github.com/wikipathways/wprdf2cytoscape)
- Active module analysis: Cytoscape (version 3.7.0), jActiveModules app (version 3.2.1)

## 3. Results

### Gene Expression

The total number of probes measured was 37,707 from which 29,024 could be linked to Ensembl identifiers. After merging multiple probe identifiers for the same Ensembl identifier, 19,023 unique gene identifiers remained. Differential gene expression analysis revealed 1,953 in the frontal cortex and 2,436 significantly changed genes in the temporal cortex samples of RETT syndrome patients vs. controls. Only 221 in frontal and 341 of the significantly changed genes in temporal cortex had a more than 1.5-fold increase or decrease in expression (|log2 fold change| > 0.58). In both brain regions, more genes were down-regulated in Rett syndrome patients than up-regulated, see Table 1, which matches with findings from the original publication (Lin et al., 2016).

**Table 1:** Differentially expressed genes in frontal and temporal cortex.

	**Temporal cortex down-regulated**	**Temporal cortex not changed**	**Temporal cortex up-regulated**
**Frontal cortex**	88	44	1
**down-regulated**	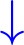 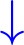	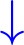 -	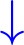 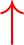
**Frontal cortex**	171	18,576	55
**not changed**	- 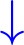	- -	- 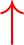
**Frontal cortex**	3	62	23
**up-regulated**	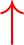 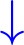	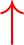 -	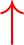 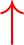

*133 and 88 genes were significantly down- and up-regulated in frontal cortex, respectively. Two hundred sixty-two and 79 genes were significantly down- and up-regulated in temporal cortex, respectively. Eighty-eight genes are down-regulated, and 23 genes are up-regulated in both brain regions. Only four genes show different expression patterns. The following filtering criteria were used: *p*-value < 0.05 and absolute log2 fold change > 0.58*.

### Gene Ontology Analysis

Gene Ontology overrepresentation analysis identified 39 and 50 biological processes as altered in frontal and temporal cortex, respectively (Supplementary Tables 2, 3). Summarizing, neuron specific and immune system-related processes were found to be enriched in both brain regions for Rett syndrome patients. In temporal cortex, additionally, regulation of translational initiation (GO:0006446) and an extracellular matrix/cytoskeleton-related process (GO:0007229) were found to be enriched. Interestingly, the microglia relevant complement factors C1QB and C1QC were found in the enriched GO classes defense response (GO:0006952) and immune effector process (GO:0002252).

### Pathway Analysis

**Figure 2:**
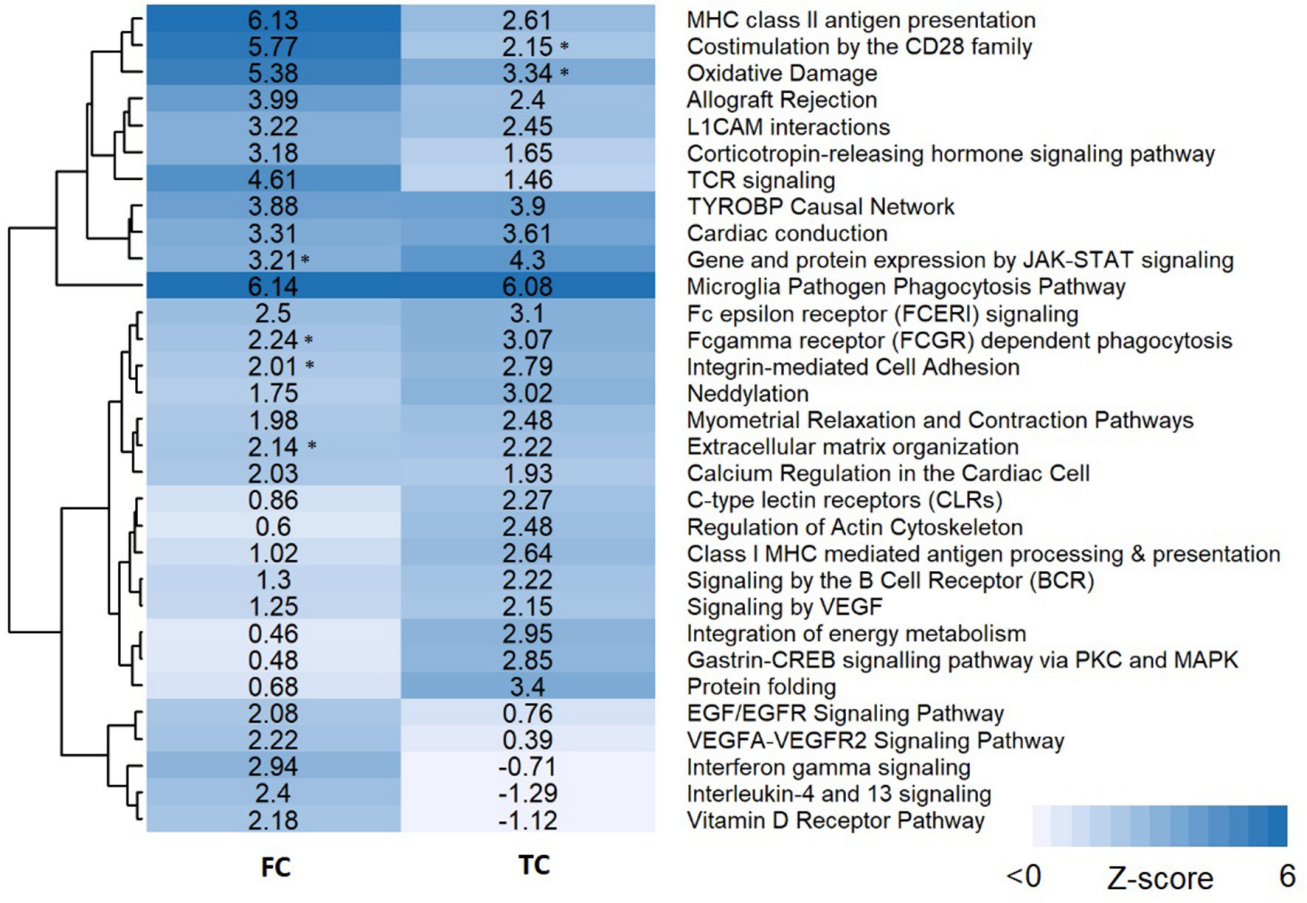
Pathway analysis results for frontal and temporal cortex data. Pathways are clustered in this heatmap based on their Z-scores. Pathways with a high Z-score (>1.96) contain significantly more changed genes than expected and are considered pathways of interest. An asterisk next to the Z-score value indicates pathways with a significant Z-score (>1.96) but less than five changed genes.

**Figure 3:**
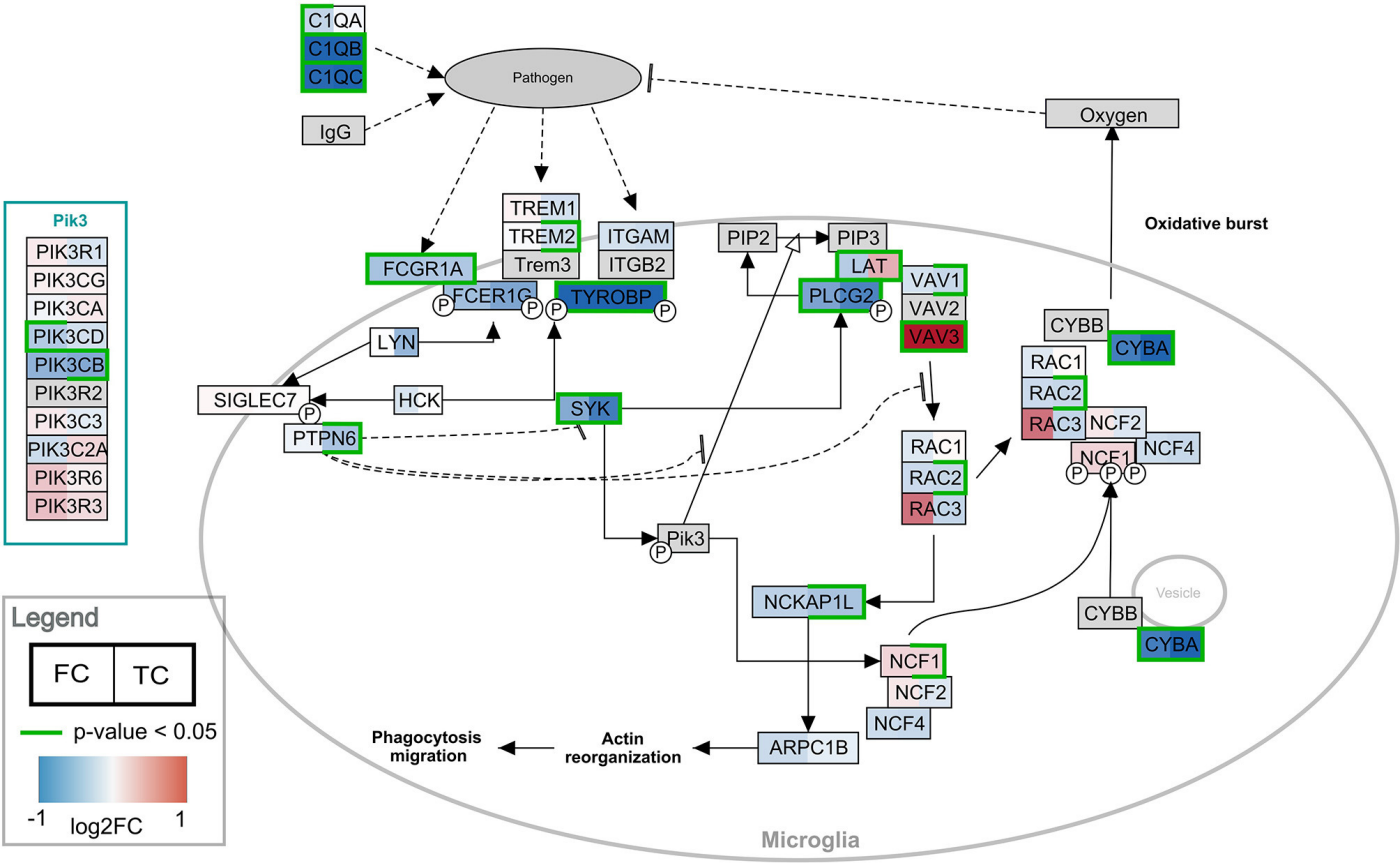
Visualization of the frontal and temporal cortex gene expression on the Microglia Pathway Phagocytosis Pathway. In the left half of the gene boxes, the gene expression change in the frontal cortex is shown. In the right half of the gene boxes, the gene expression in the temporal cortex is shown. The blue colors represent down-regulation of the gene in Rett syndrome patients (negative log2 fold change), while the red shades visualize the up-regulated genes. The darker the color, the stronger the effect. Green borders indicate significance of the change (*p*-value < 0.05). Gray colored nodes are not annotated or measured in the dataset.

Pathway analysis was performed in PathVisio for both brain regions separately. Overrepresentation analysis revealed 18 and 21 pathways altered in the datasets for frontal and temporal cortex, respectively (Z-score > 1.96, minimum five changed genes), see Figure 2. Interestingly, eight pathways were altered in both frontal and temporal cortex. Similar to the results of the GO analysis, several immune system-related and extracellular matrix/cytoskeleton-related pathways were found to be enriched. Additionally, calcium channel related processes including muscle contraction pathways were found in both brain regions. Although muscle contraction pathways are not expected in brain tissue samples, the overlapping differentially expressed genes were mostly ion channels and signaling cascade proteins also highly relevant for neurons. Supplementary Figure 1 shows the heatmap with a more lenient filter (Z-score > 1.96, minimum three changed genes). Figure 3 is an example pathway visualization for a pathway that has a high Z-score in both tissue types, Microglia Pathogen Phagocytosis Pathway (Hanspers and Slenter, 2017).

### Pathway-Based Network Construction

From the 903 pathway models in the WikiPathways and Reactome collection, 860 pathways contained 27,410 unique interactions. On average, a pathway contained 35 interactions (min = 1, max = 510, median = 22). Interestingly, 3,264 interactions occur multiple times but only 2,103 interactions are present in more than one pathway. As an example, one of the highest occurring interactions is the complex binding of the three subunits of the IκB kinase complex which plays an important role in the propagation of cellular response to inflammation (Häcker and Karin, 2006) and is present in 25 different pathways.

The resulting network consists of 48,639 nodes and 106,137 edges. The network consists of one major component (46,756 nodes) and 427 smaller components with each less than twenty nodes. The network contains 8,643 gene products, 2,704 metabolites and 9,882 complex / group nodes. Most common interaction types are directed interaction (13,572), complex / group participation (5,298), catalysis (4,787), inhibition (1,185), and conversions (896).

### Active Module Analysis

**Figure 4:**
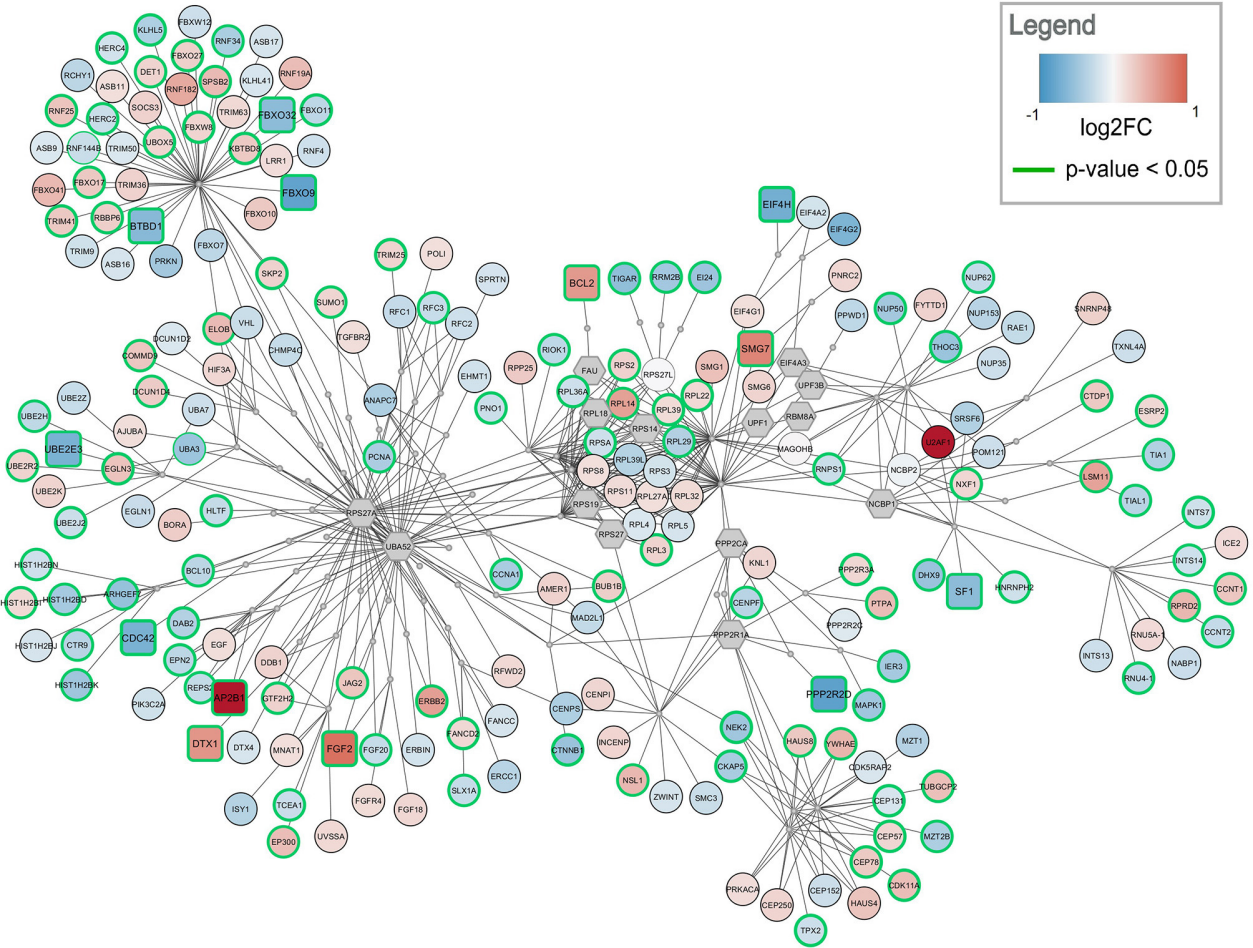
Top-ranked active module for frontal cortex data. The highest-ranked subnetwork contains 303 nodes and 568 edges. It contains 13 significantly changed genes (rounded rectangles) when applying the same cutoff as for enrichment analysis (absolute log2 fold change > 0.58). Other measured gene products are visualized as circular nodes. Blue fill color indicates down-regulation while red indicates up-regulation. The darker the color, the stronger the effect. Gray hexagons are gene products not measured in the data set. The very small, gray nodes represent interaction nodes. These were combined from 47 different pathways, with none of the pathways providing more than six interactions.

**Figure 5:**
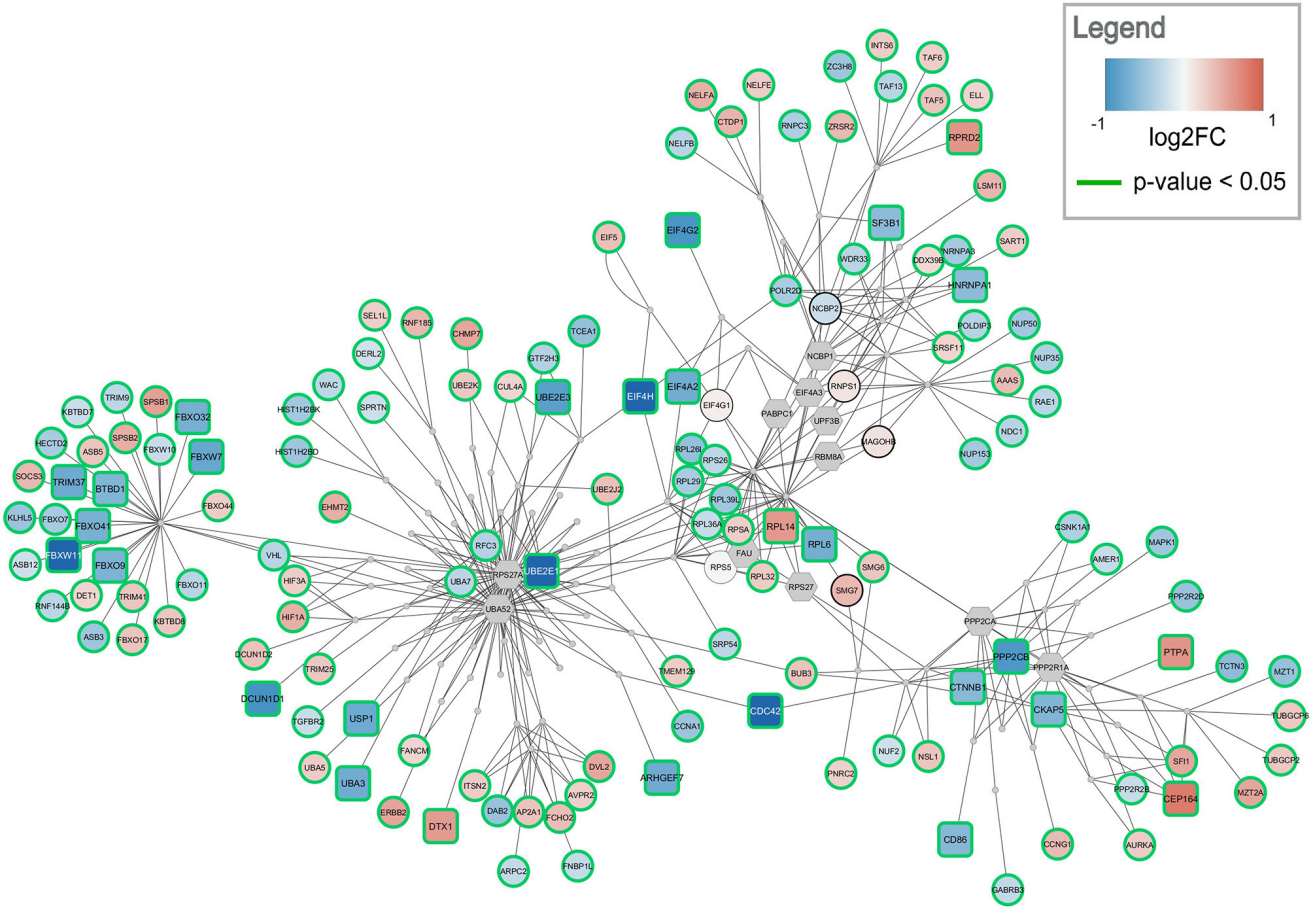
Top-ranked active module for temporal cortex data. The subnetwork contains 238 nodes and 457 edges. It contains 29 significantly changed genes (rounded rectangles) when applying the same cutoff as for enrichment analysis (absolute log2 fold change > 0.58). Other measured gene products are visualized as circular nodes. Blue fill color indicates down-regulation while red indicates up-regulation. The darker the color, the stronger the effect. Gray hexagons are gene products not measured in the data set. The very small, gray nodes represent interaction nodes. These were combined from 51 different pathways, with none of the pathways providing more than six interactions.

Active modules were calculated using the jActiveModules app. The top five modules with the highest active paths scores were identified for both comparisons, frontal and temporal cortex. The modules for frontal cortex contained between 300–350 nodes and 560–1,020 edges. The top modules for temporal cortex tended to be smaller ranging from 230–290 nodes and 450–1,000 edges. Figures 4, 5 show the highest-ranked module for frontal and temporal cortex, respectively. Gene expression changes are visualized as node color and significance is indicated by the node border color. All modules only contained gene products; no metabolites were found. The complete submodule analysis results for both datasets can be found in Supplementary Data 1 (zip file containing two Cytoscape session files).

The highest ranked active module for frontal cortex contains 303 nodes (79 interactions and 224 gene products) and 568 edges, see Figure 4. Two hundred and ten of the gene products are measured in the dataset and 112 are changed significantly (*p*-value < 0.05). Twelve gene products have an absolute log2 fold change > 0.58. The subnetwork contains eight significantly down-regulated genes (blue rounded rectangles) including two F-Box genes, *FBOX32* and *FBXO9*, involved in phosphorylation-dependent ubiquitination. The subnetwork contains five significantly up-regulated genes (red rounded rectangles) with diverse roles. The genes identified as hubs in the active module network of frontal cortex are two gene products which are not measured in the dataset, *RPS27A* and *UBA52*. Both are involved in protein degradation via 26S proteasome, ubiquitination, translation, and DNA excision repair. In the central part of the network, the ribosomal proteins including *RPL14, RPL29*, and *RPL3* form a cluster. This cluster is connected via *PPP2CA* and *PPP2R1A*, two phosphatases involved in cell cycle, DNA replication and transcription, to a cluster of centrosomal proteins including *CEP78, CEP57*, and *CEP131*. The module combines interactions from 47 unique pathways (Supplementary Table 4) including class I MHC mediated antigen processing and presentation (WP3577), non-sense-mediated decay (WP2710), cell-cycle related pathways (WP1859, WP1775, WP1858, WP2772), and eukaryotic translation elongation and initiation (WP1811, WP1812).

The highest ranked active module for temporal cortex contains 238 nodes (84 interactions and 154 gene products) and 457 edges, see Figure 5. The module partially overlaps with the module found for frontal cortex. One hundred and fourty three of the gene products are measured in the dataset and 137 are changed significantly (*p*-value < 0.05). Twenty-nine gene products have an absolute log2 fold change > 0.58. The module contains 24 significantly down-regulated genes (blue rounded rectangles) including several ubiquitin conjugating enzymes (*UBE2E1, UBE2E3*) and translation initiation factors (*EIF4A2, EIF4H, EIF4G2*). Only five significantly up-regulated genes are found in the subnetwork (red rounded rectangles) but the distance between them is large. This subnetwork contains similar hub nodes as in the frontal cortex subnetwork including *RPS27A, UBA52*, and *PPP2R1A*. Additionally, *NCBP2* and *NCBP1*, proteins involved in RNA processing, play an important role in the subnetwork. The module combines interactions from 51 unique pathways (Supplementary Table 5) including transcription / translation (WP1889, WP1906, WP1812), cell cycle (WP1859, WP1775, WP4109), and immune response (WP3577, WP2658) related processes.

## 4. Discussion

MECP2 is a multifunctional protein which is involved in several transcriptional inhibitory and activational processes. MECP2 was generally regarded as a repressor, however its role as genetic activator has also been confirmed (Chahrour et al., 2008). In previous studies, a loss of function in MECP2 due to a mutation has been found to influence a variety of pathways and biological processes, including pathways related to not only neuron development and function, but also to the immune system, transcription, and translation related processes (which were identified mainly by transcriptome analysis, Colantuoni et al., 2001; Bedogni et al., 2014; Ehrhart et al., 2018; Shovlin and Tropea, 2018). The affected pathways identified with our study closely match the results previously found by Ehrhart et al. (2018), in which human brain tissue data of Rett syndrome patients (published by Deng et al., 2007) was analyzed. The expression of the MECP2 protein itself is not significantly affected in this dataset (minor, insignificant down regulation, log2 fold change of –0.1, in both brain regions).

The original study by Lin et al. (2016) from which the dataset analyzed in this paper was acquired, focused on the significant down-regulation of certain complement system factors in Rett syndrome (C1QA, C1QB, C1QC). Complement system factors are produced generally in liver, however their expression was also found to be changed in stimulated microglia. Furthermore, there is emerging evidence that C1Q factors are involved in several non-immunogenic activities, such as synaptic pruning in microglia (Kouser et al., 2015).

As expected, our pathway and GO analysis revealed a substantial number of immune system related pathways to be affected in Rett syndrome frontal and temporal cortex tissue samples. Inflammatory processes have been identified previously in Rett syndrome patients, mouse models and *in vitro* systems, and are suspected to contribute to the development of Rett syndrome (De Felice et al., 2016; Ehrhart et al., 2018). Figure 2 shows many of affected pathways in both frontal and temporal cortex, with similar results found by GO analysis. Interestingly, no complement system or transcription / translation related pathways show up (except Microglia Pathogen Phagocytosis Pathway, which includes C1Q factors). Only seven of the 31 pathways found through pathway analysis contribute interactions to the active modules identified for frontal and temporal cortex. The modules mainly contained interactions from transcription / translation and cell cycle related pathways, which were not found with the classical enrichment analysis. These processes were also previously found in transcriptome pathway analysis of Rett syndrome (Bedogni et al., 2014; Ehrhart et al., 2018). Not surprisingly, the subnetworks do not contain metabolic reactions. Only metabolites connecting at least two genes affected by MECP2 would be present in an active subnetwork. The enrichment analysis did not show any metabolic processes that are affected, which is in line with the manifestation of Rett syndrome. Overall, the regulatory effects of MECP2, especially on DNA maintenance, cell cycle, transcription, and translation, is more prominently shown in the active modules, while immune system related responses are more present in pathway analysis. Importantly, the active module approach does not replace analyses like classical enrichment analysis but augments it. When running the active module analysis on the same network using the dataset with permuted gene labels, the resulting subnetworks are very different from the identified Rett subnetworks. This basic computational validation further strengthens our confidence that we indeed have subnetworks specific and strongly affected in Rett syndrome patients. The results of the permutation analysis are summarized in Supplementary Data 2.

This was the first time the entirety of the WikiPathways knowledgebase, including Reactome pathways, has been used to create a comprehensive human pathway-based network for network analysis of transcriptomics data. While the pathway content of both databases overlaps, both resources also contain unique information. By building a network out of pathways from a combination of pathway databases, a more complete biological (and therefore genome) coverage is enabled. Identifying active modules from a large network has some major benefits, such as the easy applicability to any gene expression dataset, ignoring predefined boundaries used in traditional pathway diagrams, and incorporating the relations and overlap between the pathways. Additionally, this method does not require researchers to predefine a certain cutoff, since genes are ranked based on their significance.

Some considerations arose when constructing and analyzing the network. For instance, some common metabolites like ATP, ADP, or NADH, while biologically necessary, were excluded from the network, since their involvement in a multitude of interactions created links between almost every node. Additionally, this approach is strongly depending on the a priori input of pathway data in terms of coverage and quality. Currently, human pathway databases contain a little over 50% of the protein coding genes (Slenter et al., 2017), which is also a probable number for the coverage of metabolites and interactions. Pathway models generally contain information about directionality of the interactions. However, available active subnetwork analysis methods only take topology but not directionality into account. This could strongly affect the identification of active submodules and would be an important extension of existing algorithms.

The active module discovery approach should be considered as an additional step after classical enrichment analysis. In this study, we used human brain transcriptomics data from a study with Rett syndrome patients, however our approach is not unique to this application or rare diseases. These diseases are by definition less common and often less extensively studied, which may result in lower availability of specific pathway models. Nonetheless, the active module approach succeeds and shows great power for additional discoveries. While rare genetic diseases have the advantage that the causative gene is (usually) known, the resulting downstream consequences can be diverse and affect a variety of pathways. By using pathway models in an integrative network approach, further use of the invaluable resources present in pathway databases is enabled and subnetworks of interest can be retrieved based on the entire body of pathways available. Using Cytoscape allows using all available apps such as the jActiveModules app to analyse our network. Importantly, the complete interaction network of WikiPathways with 48,639 nodes and 106,137 edges can be opened and analyzed in Cytoscape, despite of the network to be too large to be visualized. The use of graph databases like Neo4j, which already have connections available to Cytoscape (cyNeo4j app, Summer et al., 2015), could be a useful addition to the approach. Importantly, as part of the systems biology cycle, advanced computational analyses like the one reported in this manuscript lead to new hypotheses and ideas for experiments, which then need to be tested and validated in a laboratory.

### Conclusion

Pathway models have proven themselves as powerful tools for biologists to describe and analyse biological processes. The collaboration between the widely-adopted pathway databases WikiPathways and Reactome and the availability of their data in RDF format allowed us to integrate a large number of pathways from both databases into one large network. This enables us to perform advanced network analyses like active submodule identification. By comparing classical enrichment methods with the active submodule identification on a Rett syndrome dataset in two different brain regions, we found that both approaches provided valuable insights into the disease. Importantly, they were strongly complementary and did not show the same results.

## Data Availability Statement

The dataset analyzed for this study can be found in the Gene Expression Omnibus (https://www.ncbi.nlm.nih.gov/geo/query/acc.cgi?acc=GSE75303).

## Author Contributions

RM and FE: data analysis, literature search; LE: data preprocessing and statistical analysis; DS: data analysis; LC: literature search; CE and EW: study design; MK: data analysis, study design, literature search. All authors contributed in writing and editing of the manuscript.

### Conflict of Interest Statement

The authors declare that the research was conducted in the absence of any commercial or financial relationships that could be construed as a potential conflict of interest.
