# Supplementary material for: Beyond Pathway Analysis: Identification of Active Subnetworks in Rett Syndrome
Source: Front Genet. 2019 Feb 21;10:59. doi: 10.3389/fgene.2019.00059 (PMC6393361; doi:10.3389/fgene.2019.00059)

Supplementary Material

Beyond pathway analysis:
Identification of active subnetworks in Rett syndrome

Ryan A. Miller, Friederike Ehrhart, Lars M.T. Eijssen, Denise N. Slenter, Leopold M.G. Curfs, Chris T. Evelo, Egon L. Willighagen and Martina Kutmon*

Corresponding Author: martina.kutmon@maastrichtuniversity.nl

**Supplementary Figure 1.** Extended pathway analysis result with minimum number of changed genes set to three instead of five.


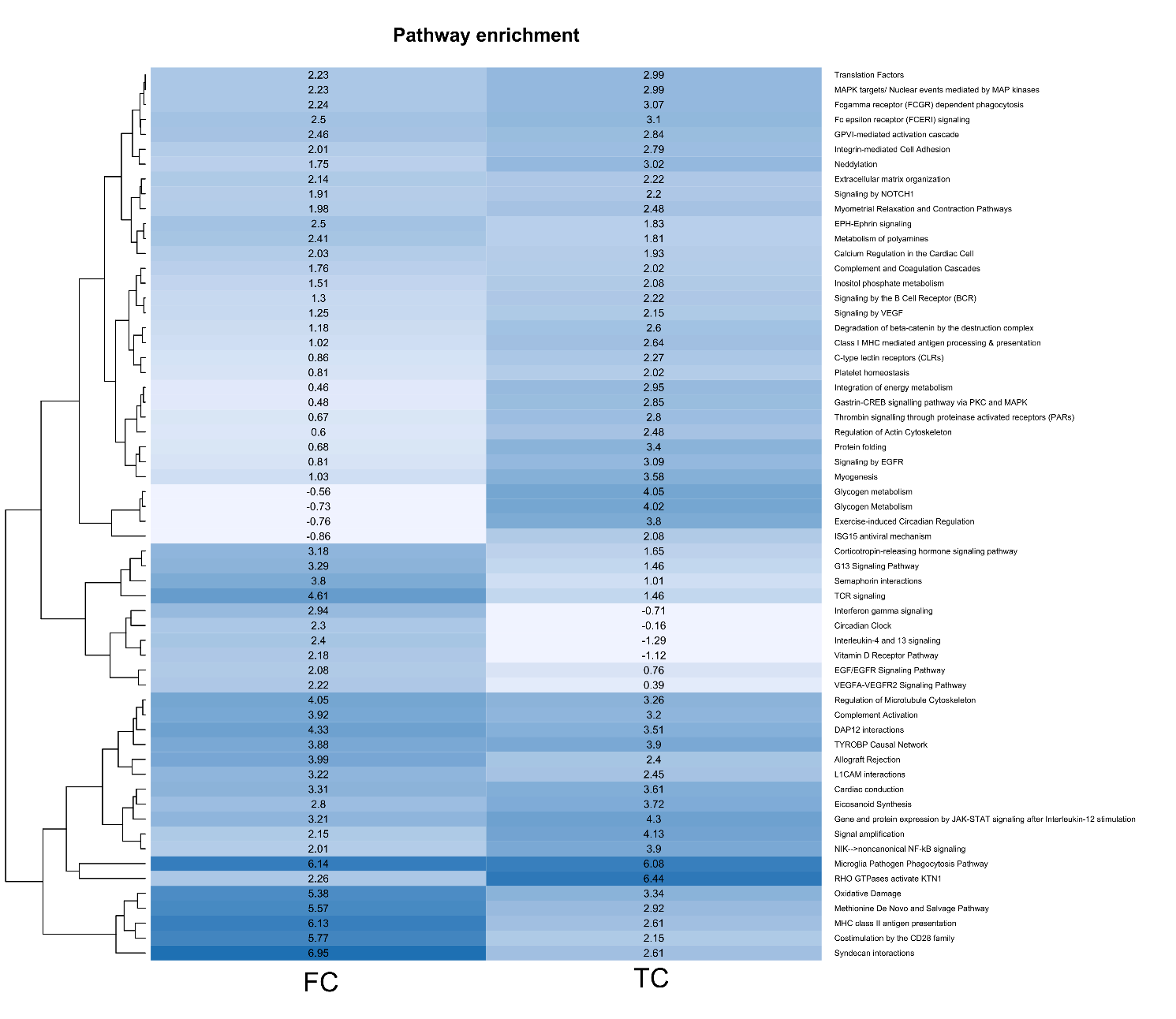


**Supplementary Table 1.** List of common metabolites excluded from the network.

| **Metabolites (Wikidata identifier + name)** | | | | | |
| --- | --- | --- | --- | --- | --- |
| Q1832 | gadolinium | Q1882 | berkelium | Q876 | selenium |
| Q428640 | unbiennium | Q7425 | unbibium | Q725 | chromium |
| Q428635 | untribium | Q428629 | untriunium | Q740 | cobalt |
| Q3551921 | unpentunium | Q869681 | untrioctium | Q1302 | flerovium |
| Q3551929 | unquadseptium | Q556 | hydrogen | Q1119 | hafnium |
| Q3552040 | untriquadium | Q1099 | antimony | Q560 | helium |
| Q1896 | fermium | Q688 | chlorine | Q629 | oxygen |
| Q1898 | mendelevium | Q1232 | dubnium | Q703 | potassium |
| Q1809 | promethium | Q1258 | meitnerium | Q713 | scandium |
| Q428692 | unbiseptium | Q1301 | nihonium | Q938 | strontium |
| Q1366419 | untriseptium | Q880 | platinum | Q682 | sulfur |
| Q2399140 | unquadennium | Q716 | titanium | Q1054 | technetium |
| Q3029913 | unquadnilium | Q1146 | unbinilium | Q1306 | tennessine |
| Q3551919 | unpentnilium | Q722 | vanadium | Q758 | zinc |
| Q3551933 | unquadtrium | Q708 | lead | Q1115 | thorium |
| Q3551935 | unquadunium | Q670 | silicon | Q650 | fluorine |
| Q1892 | einsteinium | Q932 | thallium | Q674 | phosphorus |
| Q1849 | erbium | Q1096 | tin | Q1121 | actinium |
| Q1857 | lutetium | Q663 | aluminium | Q1108 | caesium |
| Q1853 | thulium | Q706 | calcium | Q1396 | europium |
| Q1466 | unbiunium | Q671 | francium | Q861 | gallium |
| Q428685 | unbipentium | Q660 | magnesium | Q744 | nickel |
| Q22313918 | unpentquadium | Q731 | manganese | Q751 | osmium |
| Q1876 | curium | Q1252 | hassium | Q737 | rhenium |
| Q1905 | lawrencium | Q1053 | molybdenum | Q1100 | tellurium |
| Q428703 | unbioctium | Q1046 | niobium | Q1098 | uranium |
| Q3551918 | unpentbium | Q1089 | palladium | Q871 | arsenic |
| Q3551927 | unquadbium | Q677 | iron | Q1112 | barium |
| Q3551926 | unquadhexium | Q618 | boron | Q569 | beryllium |
| Q3551931 | unquadquadium | Q895 | rubidium | Q942 | bismuth |
| Q3552038 | untripentium | Q1090 | silver | Q1249 | bohrium |
| Q22314315 | unpentpentium | Q696 | argon | Q623 | carbon |
| Q1901 | nobelium | Q879 | bromine | Q1278 | copernicium |
| Q548592 | untriennium | Q1091 | cadmium | Q925 | mercury |
| Q1195471 | neutronium | Q753 | copper | Q627 | nitrogen |
| Q3552041 | untritrium | Q1266 | darmstadtium | Q1109 | protactinium |
| Q1855 | Ytterbium | Q867 | germanium | Q658 | sodium |
| Q1843 | dysprosium | Q1094 | indium | Q1385 | cerium |
| Q1846 | holmium | Q888 | krypton | Q1307 | oganesson |
| Q1819 | samarium | Q1304 | livermorium | Q979 | polonium |
| Q1838 | terbium | Q1388 | neodymium | Q1133 | radon |
| Q54377 | unbihexium | Q1105 | neptunium | Q1087 | rhodium |
| Q428648 | untrinilium | Q1102 | plutonium | Q1234 | seaborgium |
| Q3551928 | unquadoctium | Q1272 | roentgenium | Q1123 | tantalum |
| Q3551930 | unquadpentium | Q1086 | ruthenium | Q877 | iridium |
| Q1872 | americium | Q1226 | rutherfordium | Q568 | lithium |
| Q1888 | californium | Q743 | tungsten | Q1303 | moscovium |
| Q1801 | lanthanum | Q1038 | zirconium | Q1386 | praseodymium |
| Q428741 | unbiquadium | Q897 | gold | Q1128 | radium |
| Q428758 | unbitrium | Q1103 | iodine | Q1139 | ununennium |
| Q868375 | untrihexium | Q654 | neon | Q1106 | xenon |
| Q3551924 | unpenttrium | Q999 | astatine | Q941 | Yttrium |
| **Cations** | | **Anions** | |  |  |
| Q27104698 | aluminum cation | Q27110008 | sulfide(.1-) |  |  |
| Q26987404 | magnesium cation | Q29004 | hydrogen anion |  |  |
| Q27115726 | magnesium(1+) | Q417116 | telluride(2-) |  |  |
| Q27113880 | silicon(4+) | Q27113570 | telluride(.1-) |  |  |
| Q27104738 | vanadium(3+) | Q415296 | arsenide(3-) |  |  |
| Q152763 | lithium cation | Q56250422 | sulfide(2−) |  |  |
| Q27113833 | tungsten(4+) |  |  |  |  |
| Q23905776 | calcium(2+) |  |  |  |  |
| Q27104290 | strontium cation |  |  |  |  |
| Q27255903 | magnesium cation mg-28 |  |  |  |  |
| Q3154110 | sodium ion |  |  |  |  |
| Q27104730 | rubidium cation |  |  |  |  |

**Supplementary Table 2.** Pruned Gene Ontology enrichment results from GO-Elite analysis for frontal cortex.

| GO term | GO ID | Z Score | p-value | significantly changed genes |
| --- | --- | --- | --- | --- |
| response to external stimulus | GO:0009605 | 5.84 | 0.0001 | AIF1\|AP2B1\|BCL2\|BMPR1B\|CACNA1H\|CCL3L3\|CDC42\|  CDKN2D\|CHL1\|CMTM7\|CNTN1\|CSF3R\|CTSH\|CX3CR1\|  CYBA\|EXT1\|FGF2\|FKBP1B\|FSTL1\|G6PD\|HLA-DRB1\|KAL1\|  LIMK1\|NPPA\|NR4A2\|OPN3\|PKD2L1\|RBM4\|RND1\|SCN3B\|  SDC4\|SRGAP1\|SSTR1\|TNC\|TYROBP\|VAV3 |
| multicellular organismal response to stress | GO:0033555 | 5.82 | 0.0001 | BCL2\|CRH\|GABRA5\|NR4A2\|P2RX2\|TAC1 |
| antigen processing and presentation | GO:0019882 | 4.90 | 0.0001 | AP2B1\|CTSH\|CYBA\|HLA-DMB\|HLA-DPA1\|HLA-DRB1\|PSMA1\|  PSMC4\|RAB3B |
| cellular protein complex assembly | GO:0043623 | 4.89 | 0.0001 | AIF1\|CYBA\|GREM1\|HCLS1\|HIP1\|HLA-DMB\|HSPA4\|RAP1GDS1\|  TCP1\|VAMP1 |
| intracellular signal transduction | GO:0035556 | 4.63 | 0.0001 | AIF1\|ARL17A\|ARL17B\|ARL4A\|BCL2\|CCM2\|CD86\|CDC42\|CTSH\|  DOCK9\|DUSP6\|FGF2\|FKBP1B\|GNA15\|HCLS1\|LIMK1\|MAPK10\|  NDST1\|P2RY12\|PI4KA\|PLCD1\|PLCG2\|PRKCH\|PSMA1\|PSMC4\|  RAB3B\|RBM4\|RND1\|S100A1\|SRGAP1\|SSTR1\|STAC2\|STMN1\|  TYROBP\|VAV3\|ZAK |
| positive regulation of lymphocyte proliferation | GO:0050671 | 4.51 | 0.0005 | AIF1\|BCL2\|CD86\|HLA-DMB\|HLA-DPA1\|TAC1\|VAV3 |
| positive regulation of transport | GO:0051050 | 4.49 | 0.0005 | C3\|CACNA1H\|CDC42\|CNTN1\|CRH\|GREM1\|HCLS1\|HIP1\|HLA-DRB1\|  MLC1\|NMU\|P2RX2\|P2RY12\|PLCG2\|RAB3B\|SCN3B\|SYTL4\|TAC1 |
| defense response | GO:0006952 | 4.13 | 0.0001 | AIF1\|ALOX5\|APOL2\|BCL2\|BMPR1B\|C1QB\|C1QC\|C3\|CCL3L3\|CD86\|  CDC42\|CRH\|CSF3R\|CX3CR1\|CYBA\|DUSP6\|FBXO9\|FGF2\|GABRA5\|  HLA-DRB1\|KLRC2\|LIMK1\|LY86\|MAPK10\|MLF2\|NDST1\|PLCG2\|TAC1\|  TYROBP\|VAV3 |
| immune effector process | GO:0002252 | 3.98 | 0.0001 | BCL2\|C1QB\|C1QC\|C3\|CD86\|CTSH\|CX3CR1\|HLA-DRB1\|PLCG2\|RNF8\|  RORA\|TYROBP |
| ion transmembrane transport | GO:0034220 | 3.78 | 0.0001 | ATP1B3\|ATP2B1\|ATP2C1\|CACNA1H\|FKBP1B\|GABRA5\|GLRA2\|  KCNN3\|KCNS3\|KCNV1\|P2RX2\|P2RY12\|PKD2L1\|RAP1GDS1\|SCN1A\|SCN3B |
| regulation of metal ion transport | GO:0010959 | 3.66 | 0.001 | BCL2\|CNTN1\|CRH\|FKBP1B\|P2RX2\|P2RY12\|PLCG2\|SCN3B |
| regulation of intracellular transport | GO:0032386 | 3.54 | 0.0005 | CABP1\|CDC42\|FAF1\|FKBP1B\|GREM1\|HCLS1\|MAP1B\|MLC1\|RBM4 |
| sulfur compound metabolic process | GO:0006790 | 3.37 | 0.001 | AMD1\|EXT1\|G6PD\|GAL3ST4\|MAT2B\|NDST1\|SDC4\|SPOCK3 |
| negative regulation of cell migration | GO:0030336 | 3.31 | 0.004 | AIF1\|BCL2\|CX3CR1\|FGF2\|GREM1\|KRT16 |
| cellular response to hormone stimulus | GO:0032870 | 3.28 | 0.001 | AIF1\|AP3S1\|CACNA1H\|CRH\|CTSH\|FBXO32\|FGF2\|GRB10\|NR4A2\|  RORA\|SSTR1\|TNC |
| positive regulation of angiogenesis | GO:0045766 | 3.23 | 0.003 | C3\|CTSH\|CX3CR1\|FGF2\|GREM1 |
| cell communication | GO:0007154 | 3.13 | 0.0001 | AIF1\|AP2B1\|BCL2\|CD86\|CDC42\|CRH\|FGF2\|GABRA5\|GAL3ST4\|  GJB6\|GREM1\|ITGB4\|KCNN3\|KCNS3\|KCNV1\|NR4A2\|P2RX2\|SSTR1\|  TAC1\|TNC\|VAMP1 |
| response to organic cyclic compound | GO:0014070 | 3.09 | 0.0005 | AIF1\|BCL2\|CRH\|CYBA\|DUSP6\|G6PD\|NFIA\|TAC1 |
| actin filament organization | GO:0007015 | 3.07 | 0.005 | AIF1\|BCL2\|CDC42\|HCLS1\|RND1 |
| potassium ion transport | GO:0006813 | 3.03 | 0.0045 | ATP1B3\|KCNN3\|KCNS3\|KCNV1\|P2RY12\|PKD2L1 |
| neuropeptide signaling pathway | GO:0007218 | 2.92 | 0.0025 | GLRA2\|GPR125\|NMU\|SSTR1\|TAC1 |
| angiogenesis | GO:0001525 | 2.87 | 0.001 | ADAM15\|CDC42\|FGF2\|GREM1\|OVOL2\|PLCD1\|RORA\|VAV3 |
| regulation of programmed cell death | GO:0043067 | 2.67 | 0.029 | AIF1\|BCL2\|CDC42\|CDKN2D\|CHL1\|CRH\|CTSH\|DUSP6\|FAF1\|  FGF2\|G6PD\|GABRA5\|HCLS1\|HIP1\|ING3\|MAEA\|NR4A2\|PLCG2\|  PRKCH\|PSMA1\|PSMC4\|VAV3\|ZAK |
| protein polyubiquitination | GO:0000209 | 2.66 | 0.006 | BCL2\|PSMA1\|PSMC4\|RNF8\|UBE2E3 |
| sensory perception of sound | GO:0007605 | 2.63 | 0.008 | CDKN2D\|GABRA5\|GJB6\|NAV2\|P2RX2 |
| amine biosynthetic process | GO:0009309 | 2.61 | 0.0065 | AMD1\|BBOX1\|CHDH\|MAT2B\|NR4A2 |
| biological adhesion | GO:0022610 | 2.57 | 0.024 | ADAM15\|AEBP1\|ATP2C1\|BCL2\|BMPR1B\|CDC42\|CHL1\|CNTN1\|  CSF3R\|CX3CR1\|ITGB4\|KAL1\|MAEA\|MAGI1\|NTM\|P2RY12\|SSPN\|TNC |
| glycosaminoglycan metabolic process | GO:0030203 | 2.51 | 0.0125 | EXT1\|FGF2\|NDST1\|SDC4\|SPOCK3 |
| nervous system development | GO:0007399 | 2.45 | 0.004 | DYRK1A\|FGF2\|LIMK1\|MAB21L2\|NAV2\|NR4A2\|SCN3B\|SEMA4B |
| regulation of blood pressure | GO:0008217 | 2.42 | 0.0085 | CRH\|CYBA\|NPPA\|P2RX2\|TAC1 |
| negative regulation of cell death | GO:0060548 | 2.27 | 0.022 | AIF1\|BCL2\|CHL1\|CRH\|CTSH\|FGF2\|GABRA5\|HCLS1\|MAEA\|NR4A2\|  PLCG2\|PRKCH\|PSMA1\|PSMC4 |
| cell junction organization | GO:0034330 | 2.23 | 0.013 | BCL2\|CCM2\|CDC42\|FRMPD2\|ITGB4 |
| locomotory behavior | GO:0007626 | 2.23 | 0.013 | CHL1\|CRH\|NAV2\|NR4A2\|SCN1A |
| regulation of Rho GTPase activity | GO:0032319 | 2.19 | 0.012 | ARHGEF25\|DOCK9\|RAP1GDS1\|SRGAP1\|VAV3 |
| extracellular matrix organization | GO:0030198 | 2.12 | 0.009 | ADAM15\|COL24A1\|EFEMP1\|FGF2\|GREM1\|ITGB4\|KLK7\|SDC4\|TNC |
| developmental growth | GO:0048589 | 2.09 | 0.0165 | ADAM15\|BCL2\|EIF4H\|MAP1B\|TNC |
| GTP metabolic process | GO:0046039 | 2.07 | 0.014 | ARL4A\|CDC42\|GNA15\|NME7\|RAB3B\|RND1 |
| epidermal growth factor receptor signaling pathway | GO:0007173 | 2.04 | 0.014 | AP2B1\|CD86\|CDC42\|EFEMP1\|FGF2 |
| regulation of ossification | GO:0030278 | 1.97 | 0.0205 | BCL2\|BMPR1B\|FGF2\|GREM1\|TAC1 |

**Supplementary Table 3.** Pruned Gene Ontology enrichment results from GO-Elite analysis for temporal cortex.

| GO term | GO ID | Z Score | p-value | significantly changed genes |
| --- | --- | --- | --- | --- |
| associative learning | GO:0008306 | 5.55 | 0.0001 | ATP1A3\|CACNA1C\|CRH\|DRD4\|GABRA5\|GRIN2A\|TAC1 |
| regulation of ion transmembrane transporter activity | GO:0032412 | 5.20 | 0.0001 | DRD4\|FGF12\|PDE4D\|PLCG2\|SCN3B |
| locomotory behavior | GO:0007626 | 4.81 | 0.0001 | ALDH1A3\|ATP1A3\|CACNA1C\|CHL1\|CRH\|DRD4\|ETV5\|FGF12\|FGF14\|GAA\|  NOVA1 |
| neutrophil chemotaxis | GO:0030593 | 4.78 | 0.001 | CSF3R\|EDN3\|PDE4D\|SYK\|VAV3 |
| regulation of translational initiation | GO:0006446 | 4.78 | 0.001 | EIF3CL\|EIF4A2\|EIF4G2\|EIF4H\|RBM4 |
| cell-cell signaling | GO:0007267 | 4.35 | 0.0001 | ADAM10\|CACNA1C\|CD86\|CDC42\|CRH\|CTNNB1\|DRD4\|EDN3\|ETV5\|FGF12\|  FGF14\|GABRA5\|GAL3ST4\|GNG10\|GRAP\|GRIN2A\|KCNAB1\|KCNCS\|KIF5A\|  NOVA1\|RIMS1\|SSTR1\|TAC1\|TNC\|USP14\|VAMP1\|VIPR2\|ZYX |
| purine-containing compound metabolic process | GO:0072521 | 4.34 | 0.0001 | ABCC12\|ACOT7\|AK5\|AMD1\|ARL4A\|ATP1A3\|CDC42\|DDX25\|EIF4A2\|GIMAP7\|  GNA15\|GNAQ\|GNG10\|GUCY1B3\|MACROD1\|MAT2B\|NME7\|NUDT9\|OGG1\|  PDE4D\|PPP2R4\|PSMC4\|RAB2A\|RAB3B\|RTEL1-TNFRSF6B\|SULT1A1 |
| microtubule-based transport | GO:0010970 | 4.26 | 0.002 | AP3S1\|CDC42\|KLC2\|MAP1B\|NME7 |
| antigen processing and presentation of exogenous antigen | GO:0019884 | 4.05 | 0.0005 | AP1S1\|CYBA\|HLA-DMB\|HLA-DPA1\|KIF5A\|KLC2\|PSMA1\|PSMC4 |
| integrin-mediated signaling pathway | GO:0007229 | 4.05 | 0.001 | ADAM10\|CCM2\|ITGB4\|SYK\|TYROBP\|VAV3 |
| stress-activated protein kinase signaling cascade | GO:0031098 | 3.69 | 0.0015 | CCM2\|DUSP6\|FGF12\|FGF14\|MAPK10\|RBM4 |
| nucleobase, nucleoside and nucleotide metabolic process | GO:0055086 | 3.68 | 0.0001 | ABCC12\|ACOT7\|AK5\|AMD1\|ARL4A\|ASPDH\|ATP1A3\|CDC42\|DDX25\|EIF4A2\|  GIMAP7\|GNA15\|GNAQ\|GNG10\|GUCY1B3\|MACROD1\|MAT2B\|NME7\|NUDT9\|  OGG1\|PDE4D\|PPP2R4\|PSMC4\|RAB2A\|RAB3B\|RTEL1-TNFRSF6B\|SULT1A1 |
| regulation of circadian rhythm | GO:0042752 | 3.65 | 0.0015 | CRH\|DRD4\|FBXW11\|OPN3\|RBM4 |
| viral reproduction | GO:0016032 | 3.61 | 0.0001 | ANKRD17\|AP1S1\|CD86\|EIF4A2\|EIF4H\|ELMO1\|FBXW7\|HNRNPA1\|  HNRNPK\|KPNA2\|PSMA1\|PSMC4\|RPL14\|RPL6\|SEPT6\|SYK\|ZYX |
| positive regulation of endothelial cell proliferation | GO:0001938 | 3.54 | 0.0015 | ACVRL1\|CYBA\|ECM1\|F3\|VIP |
| translational initiation | GO:0006413 | 3.42 | 0.0015 | EIF3CL\|EIF4A2\|EIF4G2\|EIF4H\|RPL14\|RPL6 |
| cellular protein complex assembly | GO:0043623 | 3.36 | 0.0001 | AIF1\|CADPS2\|CYBA\|EID2\|HLA-DMB\|HSPA4\|PDE4D\|RAP1GDS1\|  TCP1\|VAMP1 |
| memory | GO:0007613 | 3.34 | 0.002 | ATP1A3\|CRH\|DRD4\|GRIN2A\|PDE4D\|TAC1 |
| positive regulation of lymphocyte proliferation | GO:0050671 | 3.19 | 0.0015 | AIF1\|CD86\|HLA-DMB\|HLA-DPA1\|SYK\|TAC1\|VAV3 |
| regulation of intracellular transport | GO:0032386 | 3.15 | 0.0005 | CACNA1C\|CDC42\|DACT1\|FBXW11\|GNAQ\|LCP1\|MAP1B\|OGG1\|  PDE4D\|PPM1A\|RBM4 |
| coagulation | GO:0050817 | 3.14 | 0.0001 | ATP1B3\|ATP2B1\|CDC42\|DGKI\|DOCK9\|F3\|GNA15\|GNAQ\|GUCY1B3\|  JMJD1C\|KIF5A\|KLC2\|P2RY12\|PLCG2\|SLC7A6\|SYK\|VAV3 |
| neuromuscular process | GO:0050905 | 3.13 | 0.003 | ALDH1A3\|FGF12\|FGF14\|GAA\|GRIN2A |
| regulation of heart contraction | GO:0008016 | 3.07 | 0.004 | CACNA1C\|EDN3\|GAA\|GLRX3\|PDE4D\|SCN3B\|TAC1 |
| cellular polysaccharide biosynthetic process | GO:0033692 | 3.04 | 0.0045 | GYG2\|MAPK10\|MAT2B\|PIM1\|UGP2 |
| positive regulation of behavior | GO:0048520 | 3.03 | 0.002 | ADAM10\|AIF1\|CRH\|EDN3\|F3\|NR2C2 |
| monovalent inorganic cation transport | GO:0015672 | 3.01 | 0.0005 | ANK1\|ATP1A3\|ATP1B3\|ATP6V1G2\|KCNAB1\|KCNC2\|KCNIP4\|  P2RY12\|PKD2L1\|RAP1GDS1\|SCN2A\|SCN3B\|SLC17A1 |
| response to ethanol | GO:0045471 | 2.99 | 0.0045 | CRH\|CSF2RA\|DRD4\|GRIN2A\|OGG1\|TNC |
| intracellular protein transport | GO:0006886 | 2.85 | 0.001 | ANK1\|AP1S1\|AP3S1\|ARF4\|ARL4A\|ARL5A\|BCAP29\|C11orf73\|  ERP29\|HSPA4\|KPNA2\|RAB2A\|RAB3B\|RGPD8\|RIMS1\|RPL14\|RPL6\|ZFAND6 |
| establishment or maintenance of cell polarity | GO:0007163 | 2.82 | 0.0075 | ANK1\|CDC42\|CKAP5\|MAP1B\|NCKAP1 |
| regulation of blood pressure | GO:0008217 | 2.67 | 0.0055 | ACVRL1\|CACNA1C\|CRH\|CYBA\|EDN3\|TAC1\|WNK1 |
| regulation of cAMP biosynthetic process | GO:0030817 | 2.62 | 0.005 | CRH\|DRD4\|GNAQ\|P2RY12\|P2RY13\|VIP\|VIPR2 |
| heterocycle biosynthetic process | GO:0018130 | 2.59 | 0.0045 | AK5\|AMD1\|ANK1\|ASPDH\|ATP1A3\|GUCY1B3\|MAT2B\|MOCS2\|NME7 |
| regulation of protein stability | GO:0031647 | 2.55 | 0.0055 | CDC42\|DACT1\|FBXW11\|FBXW7\|GNAQ\|NCKAP1 |
| neuron projection development | GO:0031175 | 2.52 | 0.007 | ATCAY\|CHL1\|CNTN1\|LST1\|MAP1B\|TNC |
| energy reserve metabolic process | GO:0006112 | 2.46 | 0.0055 | CACNA1C\|GAA\|GNG10\|GYG2\|KCNC2\|UGP2 |
| positive regulation of neurogenesis | GO:0050769 | 2.41 | 0.0115 | BCL11A\|CAPRIN1\|CTNNB1\|ETV5\|MAP1B\|NPTN |
| positive regulation of cell projection organization | GO:0031346 | 2.40 | 0.007 | BCL11A\|CAPRIN1\|CDC42\|CNTN1\|MAP1B\|NCKAP1\|NPTN |
| gene expression | GO:0010467 | 2.38 | 0.0125 | BRF1\|CCNC\|EEF1B2\|EIF4A2\|EIF4H\|HNRNPA1\|HNRNPK\|MED27\|  NR2C2\|PPM1A\|PSMA1\|PSMC4\|RPL14\|RPL6\|SF3B1 |
| translation | GO:0006412 | 2.36 | 0.0085 | EEF1B2\|EIF4A2\|EIF4H\|MRPS33\|RBM3\|RPL14\|RPL6 |
| calcium ion transmembrane transport | GO:0070588 | 2.35 | 0.01 | ATP2B1\|ATP2C1\|CACNA1C\|GRIN2A\|P2RY12\|PKD2L1 |
| regulation of protein localization | GO:0032880 | 2.31 | 0.0155 | ATCAY\|CDC42\|CTNNB1\|DACT1\|DRD4\|FBXW11\|FBXW7\|GNAQ\|  LCP1\|NCKAP1\|OGG1\|PPM1A\|SYK\|VIP |
| regulation of protein catabolic process | GO:0042176 | 2.25 | 0.006 | ATG4B\|CDC42\|DACT1\|FBXW7\|GRIN2A\|USP14\|VIP |
| regulation of ion homeostasis | GO:2000021 | 2.18 | 0.011 | CACNA1C\|CTNNB1\|FGF12\|PDE4D\|PLCG2\|SCN3B\|TAC1 |
| lung development | GO:0030324 | 2.11 | 0.018 | C11orf73\|CRH\|CTNNB1\|FBXW7\|PDE4D |
| Notch signaling pathway | GO:0007219 | 2.08 | 0.0185 | ADAM10\|CCNC\|CNTN1\|DTX1\|FBXW7 |
| cell activation involved in immune response | GO:0002263 | 2.08 | 0.018 | CD86\|LCP1\|PLCG2\|SYK\|TYROBP |
| transmembrane receptor protein tyrosine kinase signaling pathway | GO:0007169 | 2.08 | 0.03 | ADAM10\|AP3S1\|ARF4\|ARHGEF7\|ATP6V1G2\|CD86\|CDC42\|  DUSP6\|FGF12\|FGFRL1\|GRB10\|PPM1A\|PPP2CB\|RTN4\|VAV3 |
| xenobiotic metabolic process | GO:0006805 | 2.03 | 0.02 | AKR1C1\|MAT2B\|SMOX\|SULT1A1\|TBXAS1\|UGP2 |
| platelet activation | GO:0030168 | 2.00 | 0.0165 | DGKI\|GNA15\|GNAQ\|P2RY12\|PLCG2\|SYK\|VAV3 |
| negative regulation of transport | GO:0051051 | 1.96 | 0.0135 | CHRFAM7A\|CRH\|DRD4\|FBXW11\|GRB10\|MAP1B\|P2RY12\|PPM1A\|VIP\|WNK1 |

**Supplementary Table 4.** List of 47 pathways contributing interactions to the top-ranked active submodule for frontal cortex.

| Pathway identifier | Pathway name | Number of interactions |
| --- | --- | --- |
| WP1980 | Nucleotide Excision Repair | 6 |
| WP1889 | Processing of Capped Intron-Containing Pre-mRNA | 5 |
| WP1859 | Mitotic G2-G2/M phases | 4 |
| WP4055 | E3 ubiquitin ligases ubiquitinate target proteins | 4 |
| WP1803 | DNA Damage Bypass | 3 |
| WP2710 | Nonsense-Mediated Decay (NMD) | 3 |
| WP3577 | Class I MHC mediated antigen processing & presentation | 3 |
| WP4109 | Regulation of mitotic cell cycle | 3 |
| WP4286 | Genotoxicity pathway | 3 |
| WP1775 | Cell Cycle Checkpoints | 2 |
| WP1812 | Eukaryotic Translation Initiation | 2 |
| WP1858 | Mitotic G1-G1/S phases | 2 |
| WP1906 | RNA Polymerase II Transcription | 2 |
| WP1910 | Signaling by EGFR | 2 |
| WP2652 | Mitotic Prometaphase | 2 |
| WP2658 | HIV Life Cycle | 2 |
| WP2720 | Signaling by NOTCH1 | 2 |
| WP2727 | Regulation of Hypoxia-inducible Factor (HIF) by oxygen | 2 |
| WP2772 | S Phase | 2 |
| WP2780 | Signaling by ERBB2W | 2 |
| WP3338 | Signaling by FGFR2 | 2 |
| WP3569 | Fanconi Anemia Pathway | 2 |
| WP3575 | Endosomal Sorting Complex Required For Transport (ESCRT) | 2 |
| WP4065 | Clathrin-mediated endocytosis | 2 |
| WP1799 | Costimulation by the CD28 family | 1 |
| WP1811 | Eukaryotic Translation Elongation | 1 |
| WP1890 | Processing of Capped Intronless Pre-mRNA | 1 |
| WP1898 | Regulation of DNA replication | 1 |
| WP1925 | Synthesis of DNA | 1 |
| WP1984 | Integrated Breast Cancer Pathway | 1 |
| WP2653 | PIP3 activates AKT signaling | 1 |
| WP2654 | Mitotic Prophase | 1 |
| WP2672 | ISG15 antiviral mechanism | 1 |
| WP2718 | Signaling by NOTCH2 | 1 |
| WP2742 | Signaling by TGF-beta Receptor Complex | 1 |
| WP2755 | Transcriptional activity of SMAD2/SMAD3:SMAD4 heterotrimer | 1 |
| WP2757 | Mitotic Metaphase and Anaphase | 1 |
| WP2773 | Degradation of beta-catenin by the destruction complex | 1 |
| WP3334 | Signaling by FGFR4 | 1 |
| WP3335 | Signaling by FGFR1 | 1 |
| WP3391 | Senescence-Associated Secretory Phenotype (SASP) | 1 |
| WP3791 | Selenoamino acid metabolism | 1 |
| WP3797 | Major pathway of rRNA processing in the nucleolus and cytosol | 1 |
| WP3827 | RNA polymerase II transcribes snRNA genes | 1 |
| WP399 | Wnt Signaling Pathway and Pluripotency | 1 |
| WP4102 | Deubiquitination | 1 |
| WP4121 | Neddylation | 1 |

**Supplementary Table 5.** List of 51 pathways contributing interactions to the top-ranked active submodule for temporal cortex.

| Pathway identifier | Pathway name | Number of interactions |
| --- | --- | --- |
| WP1889 | Processing of Capped Intron-Containing Pre-mRNA | 6 |
| WP4055 | E3 ubiquitin ligases ubiquitinate target proteins | 6 |
| WP1803 | DNA Damage Bypass | 5 |
| WP1980 | Nucleotide Excision Repair | 5 |
| WP1859 | Mitotic G2-G2/M phases | 3 |
| WP1906 | RNA Polymerase II Transcription | 3 |
| WP2710 | Nonsense-Mediated Decay (NMD) | 3 |
| WP3577 | Class I MHC mediated antigen processing & presentation | 3 |
| WP4065 | Clathrin-mediated endocytosis | 3 |
| WP4109 | Regulation of mitotic cell cycle | 3 |
| WP1775 | Cell Cycle Checkpoints | 2 |
| WP1812 | Eukaryotic Translation Initiation | 2 |
| WP1858 | Mitotic G1-G1/S phases | 2 |
| WP1910 | Signaling by EGFR | 2 |
| WP2652 | Mitotic Prometaphase | 2 |
| WP2658 | HIV Life Cycle | 2 |
| WP2727 | Regulation of Hypoxia-inducible Factor (HIF) by oxygen | 2 |
| WP2772 | S Phase | 2 |
| WP2773 | Degradation of beta-catenin by the destruction complex | 2 |
| WP2780 | Signaling by ERBB2 | 2 |
| WP3338 | Signaling by FGFR2 | 2 |
| WP3498 | Assembly of the primary cilium | 2 |
| WP3569 | Fanconi Anemia Pathway | 2 |
| WP3575 | Endosomal Sorting Complex Required For Transport (ESCRT) | 2 |
| WP4124 | Cilium Assembly | 2 |
| WP1780 | ABC-family proteins mediated transport | 1 |
| WP1799 | Costimulation by the CD28 family | 1 |
| WP1839 | Interleukin-1 family signaling | 1 |
| WP1890 | Processing of Capped Intronless Pre-mRNA | 1 |
| WP1898 | Regulation of DNA replication | 1 |
| WP1925 | Synthesis of DNA | 1 |
| WP2380 | Brain-Derived Neurotrophic Factor (BDNF) signaling pathway | 1 |
| WP2653 | PIP3 activates AKT signaling | 1 |
| WP2654 | Mitotic Prophase | 1 |
| WP2659 | Deadenylation-dependent mRNA decay | 1 |
| WP2672 | ISG15 antiviral mechanism | 1 |
| WP2720 | Signaling by NOTCH1 | 1 |
| WP2737 | SRP-dependent cotranslational protein targeting to membrane | 1 |
| WP2742 | Signaling by TGF-beta Receptor Complex | 1 |
| WP2755 | Transcriptional activity of SMAD2/SMAD3:SMAD4 heterotrimer | 1 |
| WP2757 | Mitotic Metaphase and Anaphase | 1 |
| WP3334 | Signaling by FGFR4 | 1 |
| WP3335 | Signaling by FGFR1 | 1 |
| WP3339 | TCF dependent signaling in response to WNT | 1 |
| WP3379 | RHO GTPases Activate Formins | 1 |
| WP3391 | Senescence-Associated Secretory Phenotype (SASP) | 1 |
| WP3807 | Regulation of TP53 Expression and Degradation | 1 |
| WP3827 | RNA polymerase II transcribes snRNA genes | 1 |
| WP399 | Wnt Signaling Pathway and Pluripotency | 1 |
| WP4102 | Deubiquitination | 1 |
| WP4121 | Neddylation | 1 |

**Supplementary Data 2.** Computational validation of subnetworks by creating datasets with permuted gene labels and re-running the jActiveModules analysis again.

Test 1: Permuted gene labels on the frontal cortex dataset

Resulting network contains 384 nodes (270 gene products, 4 metabolites, 110 interactions) and 702 edges. From 257 measured gene products, only 30 are significant in the original (non-permuted) dataset compared to 129 nodes in the permuted dataset. As visualized on the figure below, only very few nodes in the subnetwork are affected in Rett syndrome patients.


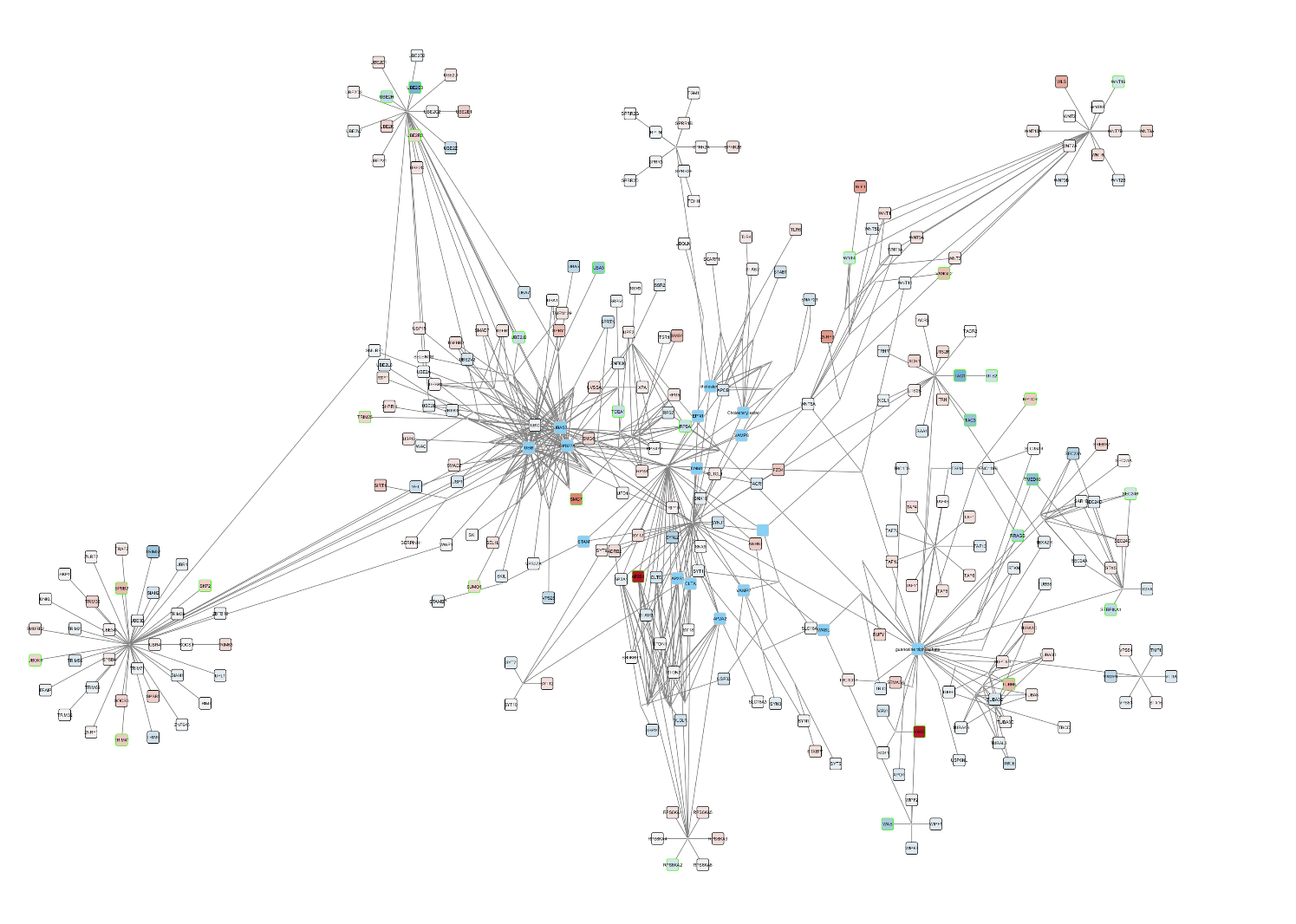


Test 2: Permuted gene labels on the frontal cortex dataset

Resulting network contains 376 nodes (267 gene products, 5 metabolites, 104 interactions) and 719 edges. From 249 measured gene products, only 29 are significant in the original (non-permuted) dataset compared to 123 nodes in the permuted dataset. As visualized on the figure below, only very few nodes in the subnetwork are affected in Rett syndrome patients.


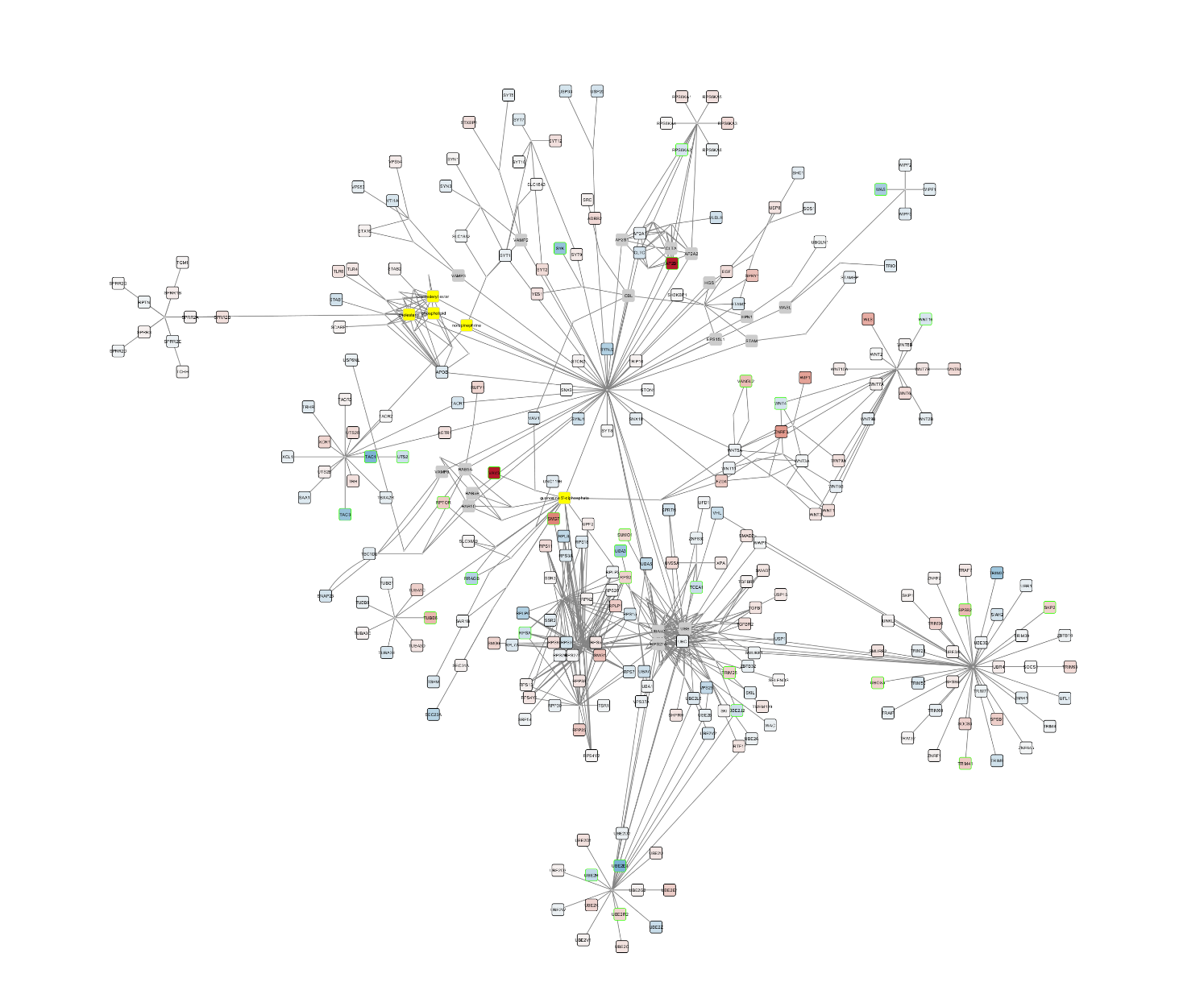


Test 3: Permuted gene labels on the frontal cortex dataset

Resulting network contains 135 nodes (134 gene products, 1 interaction) and 134 edges. From 134 measured gene products, only 13 are significant in the original (non-permuted) dataset compared to 134 nodes in the permuted dataset. Interestingly, the network represents a large Kruppel-like zinc finger protein family which showed strong differential expression in the permuted dataset. As visualized on the figure below, only very few members of the family are affected in Rett syndrome patients.


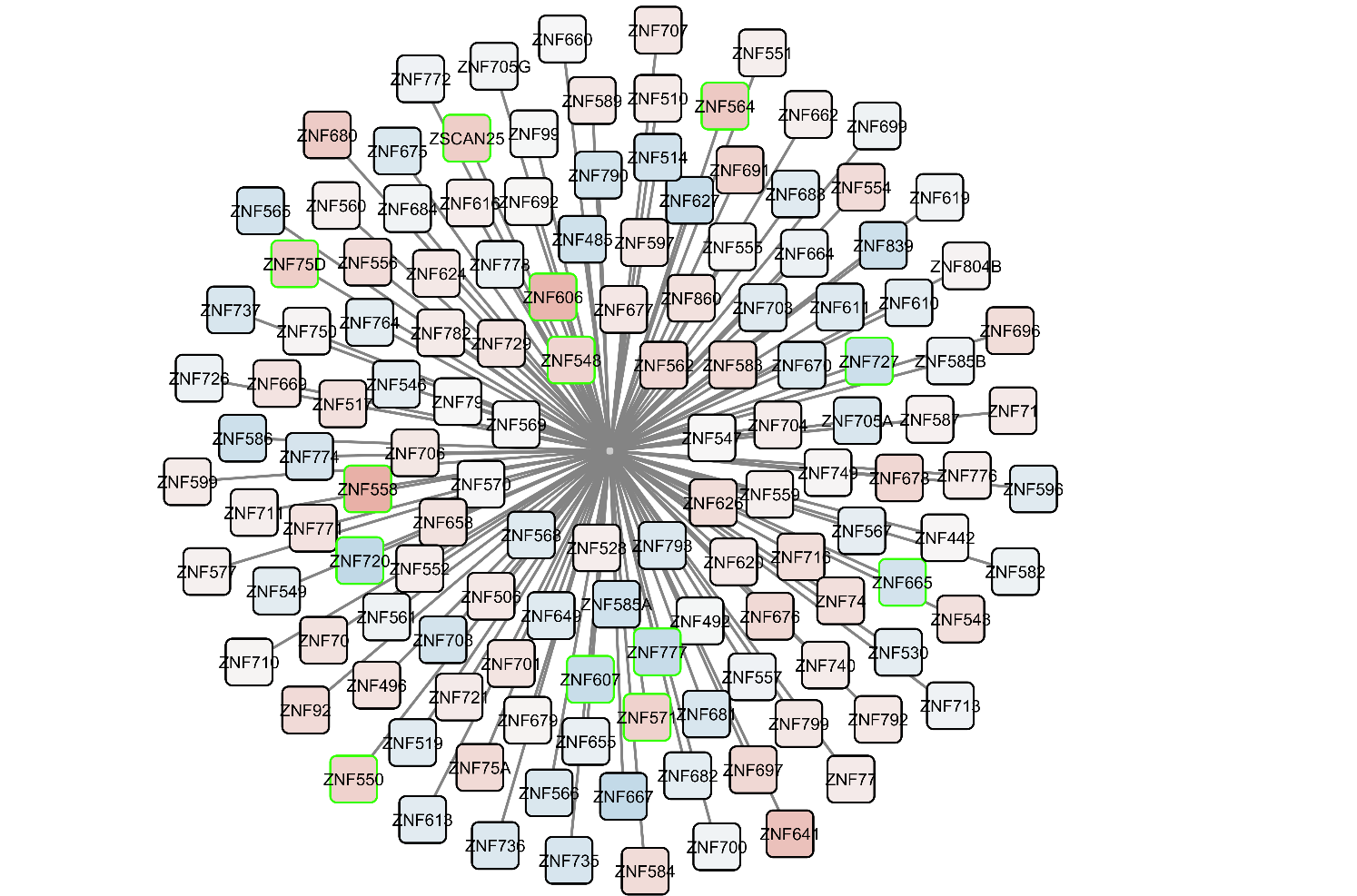

Supplement: Supplementary file 1 [file Table_1.DOCX]
